# Effects of selective and combined activation of estrogen receptor α and β on reproductive organ development and sexual behaviour in Japanese quail (*Coturnix japonica*)

**DOI:** 10.1371/journal.pone.0180548

**Published:** 2017-07-03

**Authors:** Anna Mattsson, Björn Brunström

**Affiliations:** 1 Department of Environmental Toxicology, Uppsala University, Uppsala, Sweden; 2 Centre for Reproductive Biology in Uppsala (CRU), Uppsala, Sweden; University of Texas at El Paso, UNITED STATES

## Abstract

Excess estrogen exposure of avian embryos perturbs reproductive organ development in both sexes and demasculinizes the reproductive behaviors of adult males. We have previously shown that these characteristic effects on the reproductive organs also can be induced by exposure of Japanese quail (*Coturnix japonica*) embryos to selective agonists of estrogen receptor alpha (ERα). In contrast, the male copulatory behavior is only weakly affected by developmental exposure to an ERα agonist. To further elucidate the respective roles of ERα and ERβ in estrogen-induced disruption of sexual differentiation, we exposed Japanese quail embryos *in ovo* to the selective ERα agonist 16α-lactone-estradiol (16αLE_2_), the selective ERβ agonist WAY-200070, or both substances in combination. The ERα agonist feminized the testes in male embryos and reduced cloacal gland size in adult males. Furthermore, anomalous retention and malformations of the Müllerian ducts/oviducts were seen in embryos and juveniles of both sexes. The ERβ agonist did not induce any of these effects and did not influence the action of the ERα agonist. Male copulatory behavior was not affected by embryonic exposure to either the ERα- or the ERβ-selective agonist but was slightly suppressed by treatment with the two compounds combined. Our results suggest that the reproductive organs become sexually differentiated consequent to activation of ERα by endogenous estrogens; excessive activation of ERα, but not ERβ, during embryonic development may disrupt this process. Our results also suggest that the demasculinizing effect of estrogens on male copulatory behavior is only partly mediated by ERα and ERβ, and may rather involve other estrogen-responsive pathways.

## Introduction

The sexual differentiation in birds is largely dependent on the plasma levels of gonadal estrogens; estrogens produced by the female embryo induce a female phenotype whereas the male phenotype develops at low estrogen concentrations. In Japanese quail (*Coturnix japonica*), the female embryo produces substantially higher levels of estradiol and estrone than the male embryo from at least embryonic day 5 (E5), which is at the onset of morphological sex differentiation of the gonads [1]. Treatment of female chicken and Japanese quail embryos with inhibitors of aromatase or with estrogen receptor antagonists results in partial phenotypic sex-reversal manifested as formation of testis-like gonads, development of male secondary sex characteristics, lack of oviductal development and male-like growth of the cloacal gland in response to testosterone [2–5]. Conversely, treatment of male embryos with estrogens or experimental overexpression of aromatase cause feminization of the gonads such as development of an ovary-like left testis (ovotestis) and inhibited development of the right testis (only the left ovary and oviduct develop in females) [2, 6–8]. The gonadal feminization is transient and the testicles resume a fairly normal character later in life although structural and functional effects may still be found in the adult testis [9]. In addition, the Müllerian ducts may not fully regress in estrogen-exposed male embryos and instead they develop into persisting misshapen oviduct-like structures [8, 10]. Furthermore, the cloacal gland may show reduced growth in response to circulating testosterone at sexual maturity [11]. In females, embryonic exposure to excess estrogens results in anomalous retention of the right Müllerian duct, and both Müllerian ducts may develop into oviducts that are malformed and functionally impaired in the adult hen [12, 13]. The reproductive organ abnormalities seen after embryonic exposure to excess estrogen can also be induced by *in ovo* exposure to xenoestrogens such as 1-(2-chlorophenyl)-1-(4-chlorophenyl)-2,2,2-trichloroethane (o,p’-DDT) [14, 15], ethinylestradiol (EE2) [16, 17], bisphenol A (BPA) [18] and diethylstilbestrol [16, 18].

In female quail, the brain is permanently organized by endogenous gonadal estrogens acting during a critical window of embryonic development in such a way that females are unable to display male-typical copulatory behavior in adulthood even when supplied with testosterone to adequate plasma levels [5, 19, 20]. Similar demasculinization of brain and behavior is seen also in male quail if they have been exposed to estrogenic substances *in ovo* [11, 15, 19, 21, 22]. The critical period when behavioral demasculinization can be induced in males ends before E12 [5, 11].

Endogenous estrogens and many of the known xenoestrogens act by activating either or both subtypes of nuclear estrogen receptors (ERs), α and β. The nuclear ERs function as transcription factors that upon ligand-binding form dimers that can bind to estrogen response elements of specific target genes and regulate their expression. The subtypes ERα and ERβ show differential expression patterns and may induce similar, specific or even opposing effects on gene expression and have partly different physiological roles [23, 24]. Some estrogenic compounds show preferential binding for either ERα or ERβ. For instance, BPA, genistein, coumestrol and methoxychlor show higher affinity for ERβ than ERα while EE2 and nonylphenol preferentially bind ERα [25–27]. Thus, ERα and ERβ have at least partly different biological roles and can be differentially activated by various xenoestrogens.

The temporal and spatial expression patterns of ERα and ERβ may provide insight into their respective physiological functions. In quail, ERα mRNA is expressed in left and right gonad of both sexes from at least E5, i.e. at the onset of morphological sex differentiation, and in Müllerian ducts from at least E7 [28]. In the same study, low levels of ERβ mRNA were also detected in gonads and Müllerian ducts of both sexes. In the chicken, ERα mRNA has been localized to the cortex of the left gonad and to the medulla of both gonads in both sexes just prior to and during morphological sex differentiation of the gonads [29–31]. In contrast to the dominating ERα mRNA expression in the reproductive organs, only ERβ mRNA was detected in the quail brain on E9 within brain nuclei that are implicated in the male copulatory behavior in adulthood [32]. E9 is within the sensitive period for estrogen-induced demasculinization of these behaviors [5]. Thus, the gene expression studies indicate that sexual differentiation of gonads and Müllerian ducts is mediated by ERα while demasculinization of male copulatory behaviors may predominantly be mediated by ERβ.

We have previously shown that embryonic exposure of quail to the selective ERα agonists propyl-pyrazole-triol (PPT; 300 μg/egg) and 16α-lactone-estradiol (16αLE_2_; 0.3 μg/egg) from E3 does not affect the male copulatory behavior at doses that markedly affect the reproductive organ differentiation [28, 33, 34]. Since the non-selective ER-agonists EE2 and estradiol efficiently suppressed all behaviors in the copulatory sequence at doses that caused equal or even slightly lower effect on the reproductive organs compared with the effects by PPT and 16αLE_2_, we concluded that activation of ERα alone affects the reproductive organs but is not sufficient to affect the copulatory behavior [33, 34].

Whether ERβ activation, alone or combined with ERα, affects sex differentiation of reproductive organs and copulatory behavior in Japanese quail has not been studied previously. However, in chicken the ERβ-selective agonist diarylproprionitrile (DPN) does not affect reproductive organ development, suggesting that ERβ is not involved in this process and does not mediate the effects of xenoestrogens on the reproductive organs in birds [35].

The aim of the present work was to elucidate the roles of ERα and ERβ in estrogen-induced disruption of sexual differentiation of reproductive organs and male copulatory behavior in Japanese quail. Embryos were exposed to 16αLE_2_ (ERα-selective), WAY-200070 (WAY; ERβ-selective), or both ER ligands in combination. Our results suggest that excessive activation of ERα, but not ERβ, during embryonic development disrupts sex differentiation of the reproductive organs whereas the organization of male copulatory behavior during embryo development is only weakly affected by combined activation of these receptors.

## Materials and methods

### ER-selective agonists

The ERβ selective agonist WAY (CAS number 440122-66-7; compound no 92 in Malamas et al. (2004) [36], also referred to as WAY-200070), and the ERα selective agonist 16αLE_2_ were generously provided by Wyeth Research (Collegeville, PA) and Bayer Schering Pharma AG (Berlin, Germany), respectively.

16αLE_2_ is a steroidal compound and is a close derivative of estradiol. The transactivational activity of 16αLE_2_ on human ERα (hERα) *in vitro* is 30% that of estradiol [37] and 16αLE_2_ shows a 250-fold selectivity for hERα over hERβ in reporter cells [37, 38]. WAY is a 7-substituted 2-phenyl benzoxazole [36]. Its binding affinities for human, rat and mouse ERβ are similar to those of estradiol and the selectivity for ERβ over ERα is 100-fold [36].

There are two amino acid differences between hERα and hERβ in the ligand binding pocket; Leu_384_ in hERα is replaced by Met_336_ in hERβ, and Met_421_ in hERα is replaced by Ile_373_ in hERβ [39]. The selectivity of 16αLE_2_ and WAY for ERα and ERβ, respectively, is achieved through the hERα Met_421_/hERβ Ile_373_ residue substitution [36, 37]. These residues are conserved between human and quail (S1 Fig), suggesting that 16αLE_2_ and WAY are ER-subtype specific also in quail.

### Egg incubation and exposure

The experiments and animal care were carried out according to the recommendations by the Swedish Board of Agriculture and were approved by the Uppsala Ethical Committee for Research on Animals (Uppsala district court; Permit no C198/6). The animals were euthanized quickly by decapitation, and efforts were made to minimize suffering. Animals showing signs of illness were immediately euthanized. Japanese quail eggs were purchased from Olstorp (Färgelanda, Sweden) and were placed in an egg incubator at 37°C and 60% humidity and were automatically turned every third hour. The day that the eggs were placed in the incubator was defined as embryonic day 0 (E0). Following three days of incubation, i.e. on E3, the eggs were candled and unfertilized eggs or eggs containing dead embryos were removed. Test substances were injected into the yolk of fertile eggs through a small hole made at the blunt end of the egg. The holes were then sealed with melted paraffin wax and the eggs were returned to the incubator. The injected substance is continuously taken up when the yolk is utilized. The vehicle was a peanut oil:lecithin mixture (10:1, v:w) which was emulsified in propylene glycol (1:1.5, v:v) by ultra-sonication. The injection volume was 20 μl. Egg weights were approximately 15 g. In the first experiment, eggs were injected on E3 with vehicle, 100 or 300 μg of the ERβ agonist WAY, or 0.3 μg of the ERα agonist 16αLE_2_. Embryos were dissected on E16, i.e. 1–2 days before anticipated hatching. The 300 μg-dose was not completely dissolved in the vehicle and was given as a suspension. In the second experiment, eggs were injected on E3 with vehicle, 100 μg WAY, 0.3 μg 16αLE_2_ or a combination of 100 μg WAY and 0.3 μg 16αLE_2_. Juvenile birds were examined by necropsy and adult male birds were tested regarding copulatory behavior performance before being necropsied. 16αLE_2_ was given at a dose (0.3 μg/egg) which in previous studies caused severe reproductive organ abnormalities but no effect on male copulatory behavior [33].

### Embryo dissections

In the first experiment, embryos were exposed *in ovo* to WAY or 16αLE_2_, as described in the previous section, and were dissected on E16. At this developmental stage the gonads are sexually dimorphic and the sexes are easily distinguishable in control animals. In treated embryos, sexing may be more difficult and the treated embryos were therefore also genetically sexed using a PCR-based method [40]. In each group 22 embryos were dissected, dead ones not included. All embryo dissections were performed blinded to treatment. Body length (beak to rump) was measured. The reproductive organs were examined under a stereo microscope. Frequencies of males with a left ovotestis and retained Müllerian ducts (which are normally regressed in males at this stage) were recorded. Length and width of both testes were measured *in situ* using a digital slide caliper and the testis size (length × width) was calculated. To confirm ovotestis formation, the left testis was collected and prepared for histology. In females, abnormalities of the left Müllerian duct were noted and the length of the retained part of the right Müllerian duct was measured.

### Histology of embryo testes

The left testis from the embryos was processed for histological examination as described previously [18]. Briefly, the gonads were cut in 2 μm thick sections which were collected at 60-μm intervals and stained with hematoxylin and eosin. The histology was analyzed in two transverse sections collected at a central level of the testis. The sections were photographed using a Leica Leitz DMRXE microscope equipped with a Hamamatsu ORCA III M digital camera controlled with Openlab 3.09 software from Improvision. Image analysis using ImageJ 1.36b software (Wayne Rasband, National Institute of Health, USA) was used to measure the areas consisting of cortex and medulla, respectively. The cortex area was expressed as % of total testis area in the section and was averaged for the two sections of each testis.

### Hatching and housing

In the second experiment, quail embryos were exposed *in ovo* to WAY, 16αLE_2_ or to a combination of these compounds. The birds were examined when they were juvenile (3 weeks old) or when they had reached sexual maturity (8–9 weeks old). On E14 the eggs were placed in hatching boxes, the automatic turning of the eggs was turned off and the relative humidity in the incubator was increased to 70%. The chicks hatched on days 17 to 20 of incubation, but only those hatching on days 17 to 18 were included in the experiment. Males and females were first raised together but were separated at the age of three weeks. The males were housed in individual cages from the age of four weeks. Feed (turkey starter) and water were provided *ad libitum* and the daily photoperiods were cycles of 16 h light and 8 h dark, simulating long days. All birds were weighed when they were 1 week, 2 weeks, and 3 weeks old. Males were also weighed after the behavioral tests were completed (~9 weeks old).

### Dissection of juveniles

Juvenile female and male quail were dissected at the age of 3 weeks and their reproductive organs were examined. Eleven control females and 8–9 randomly chosen males from each group were spared for behavioral tests at sexual maturity. Frequencies of males with partially developed oviduct on either side were recorded. Gonado-somatic index (GSI = 100 × testis weight / body weight) and testis weight ratio (left testis weight / right testis weight) were calculated for males. In females, oviductal abnormalities such as partial development of the right oviduct (longer than 10 mm) and malformation of the left oviduct were recorded.

### Copulatory behavior

Male sexual behaviors were recorded as described previously [21]. Briefly, each male was tested once a day for five consecutive days during the 9^th^ week after hatching. The male was paired with a sexually mature control female in a small test cage (50 × 40 cm, height 30 cm) and the behavior was observed and recorded on video for 2 minutes. Each male was paired with a new female every test occasion. The individual behaviors in the copulatory sequence that were scored were neck grab, mount attempt, mount, and cloacal contact movements, as described previously [21]. The analysis of the behaviors was performed blinded to treatment group. The number of positive trials (0–5) was counted for each bird and behavior. In addition, the mean number of times that cloacal contact movements were displayed across the five trials was determined for each bird.

### Dissections of sexually mature males

Five days after the last behavioral test (when birds were ~9 weeks old), the males were dissected and their reproductive organs were examined as described above for the juvenile males. In addition, the cloacal gland area (length × width) was recorded. Blood was collected immediately following decapitation and was centrifuged at 1000 × g. The plasma was collected and frozen at -20°C for subsequent testosterone analysis. The plasma testosterone concentration was determined using a solid phase radioimmunoassay kit (Coat-A-Count, Diagnostic Products, Los Angeles, CA) which has previously been validated for analysis of testosterone in quail plasma [21].

### Statistics

Frequencies of embryo mortality, hatching, and reproductive organ abnormalities were analyzed using one-sided Fisher's exact test. Differences in body weight, gonad size, testis weight ratio and cloacal gland area were tested using one-way analysis of variance (ANOVA). Following the ANOVA, all treated groups were compared with the control using Dunnett’s multiple comparison test. Due to non-Gaussian distributions or differences in variance between groups, testis cortex area, length of right Müllerian duct and behaviors were analyzed using Kruskal-Wallis ANOVA, followed by Dunn's multiple comparison test. The treatment groups were compared with the control. Correlation between plasma testosterone concentration and copulatory behavior was analyzed with linear regression. Differences were considered significant when p-values were below 0.05. The statistical analyses were computed using GraphPad Prism (GraphPad Software Inc, San Diego, CA, USA.).

## Results

### Embryo mortality

Mortality and beak-to-rump length by E16 and number of dissected embryos of each sex are shown in Table 1. Viability and beak-to-rump length were not affected by WAY or 16αLE_2_, suggesting lack of general toxicity at the doses tested.

**Table 1:** Doses, mortality, number of dissected 16-day-old female and male embryos, and beak-to-rump length.

Treatment Group	Dose (μg/egg)	Mortality	No. of Females	No. of Males	Beak-to-rump length (mm)#
Control	0	4/26	9	13	64 ± 2
WAY (100)	100	3/25	11	11	64 ± 3
WAY (300)	300	0/22	9	13	64 ± 2
16αLE_2_	0.3	2/24	11	11	64 ± 3

^#^Mean ± SD

### Müllerian ducts

**Fig 1:**
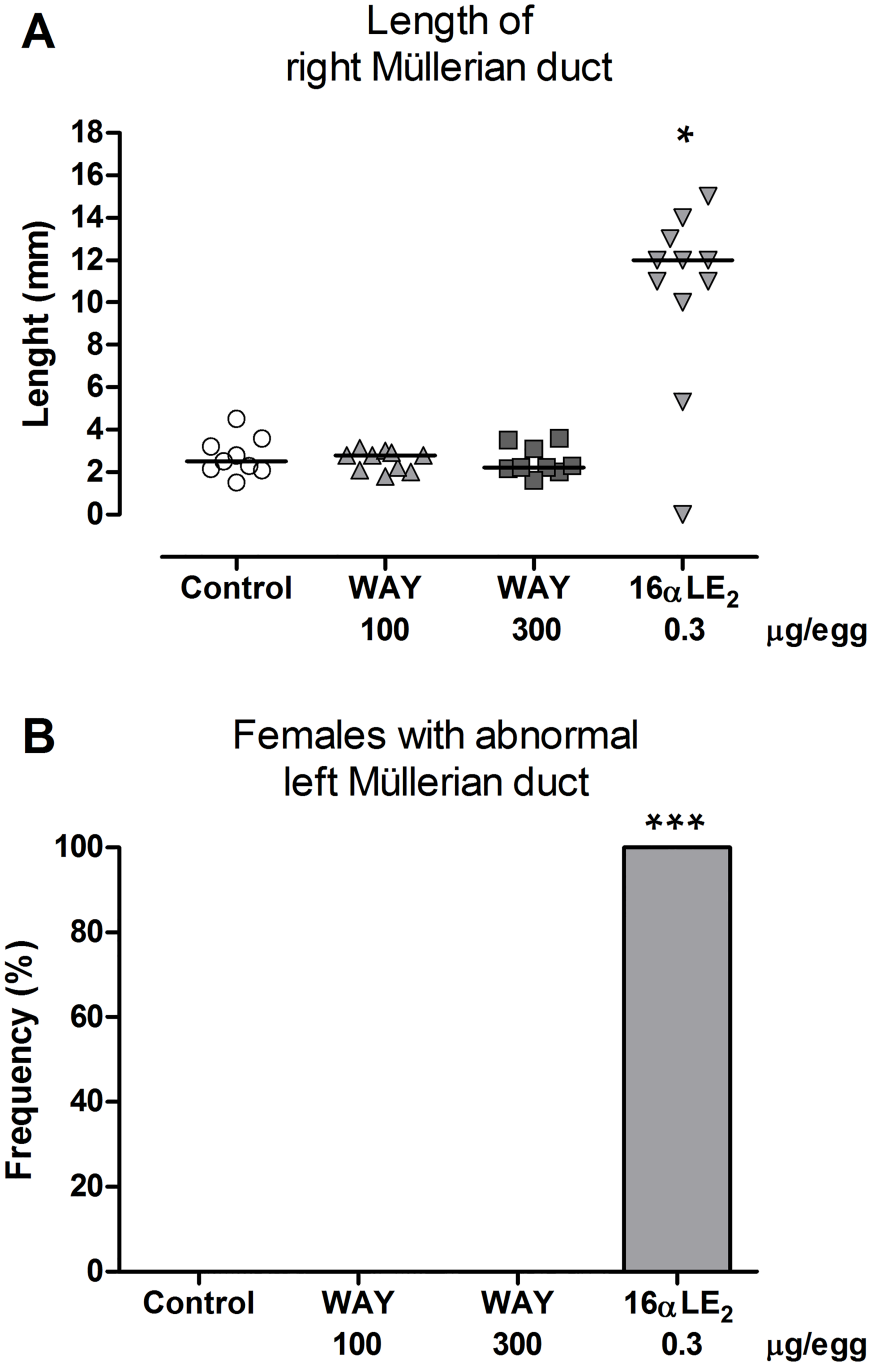
Length of right Müllerian duct (A) and frequencies of abnormal left Müllerian duct (B) in female quail embryos. Embryos were treated by *in ovo* injection on E3 with vehicle, the ERβ agonist WAY, or the ERα agonist 16αLE_2_. Effects were assessed on E16. The number of examined embryos was 9, 11, 9 and 11 in the groups from left to right in the figures. The horizontal lines in A indicate the group medians. Differences in Müllerian duct lengths were analyzed using Kruskal-Wallis ANOVA (p = 0.0014 and H = 15.6), followed by Dunn's multiple comparison test. Frequencies of abnormal ducts in the treated groups were compared with the frequency in the control using one-sided Fisher's exact test. Asterisks indicate significant differences compared with the control (*p <0.05; ***p <0.001).

The right Müllerian duct was largely regressed in female control embryos and only a few mm of the caudal part remained. The length of the right Müllerian duct was significantly increased by treatment with 16αLE_2_ (Fig 1). The left Müllerian duct was abnormal in all female embryos treated with 16αLE_2_. The duct was frequently cystic and contained a white cheese-like substance; these effects were similar to those of 16αLE_2_ described previously [33, 34]. The retained part of the right Müllerian duct showed similar malformations as the left duct. Müllerian duct gross morphology was not affected by WAY (Fig 1).

**Fig 2:**
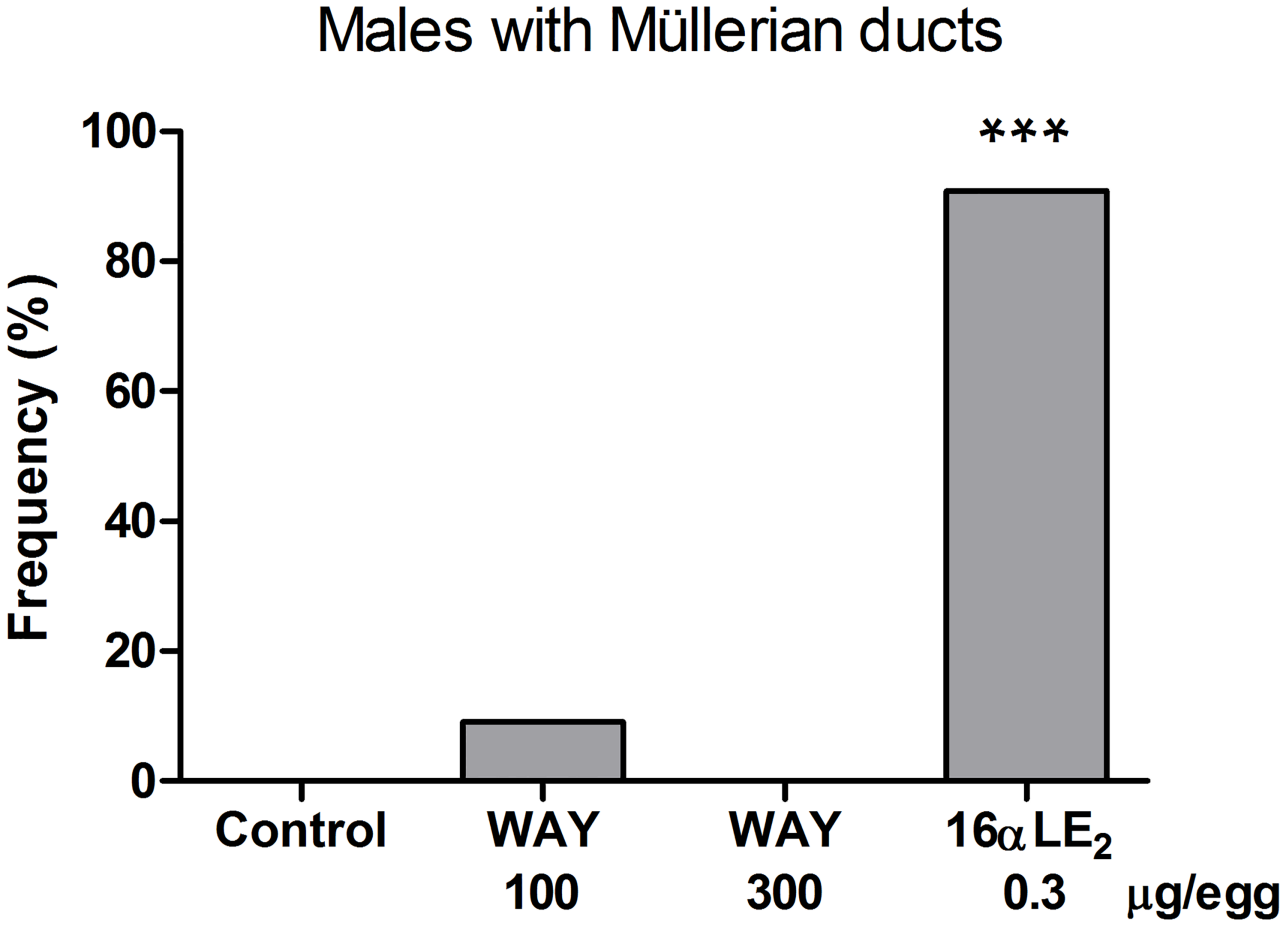
Frequencies of male quail embryos with one or both Müllerian ducts retained. Embryos were treated by *in ovo* injection on E3 with vehicle, the ERβ agonist WAY, or the ERα agonist 16αLE_2_. Effects were assessed on E16. The number of examined males was 13, 11, 13 and 11 in the groups from left to right in the figure. Frequencies of males with ducts in the treated groups were compared with the frequency in the control using one-sided Fisher's exact test (***p <0.001).

In all male control embryos, both Müllerian ducts were completely regressed. Treatment with 16αLE_2_ caused retention of one or both Müllerian ducts in ten out of eleven males (Fig 2). The retained Müllerian ducts showed similar malformations in males as described for females. WAY treatment did not induce Müllerian duct retention in male embryos.

### Embryo gonads

**Fig 3:**
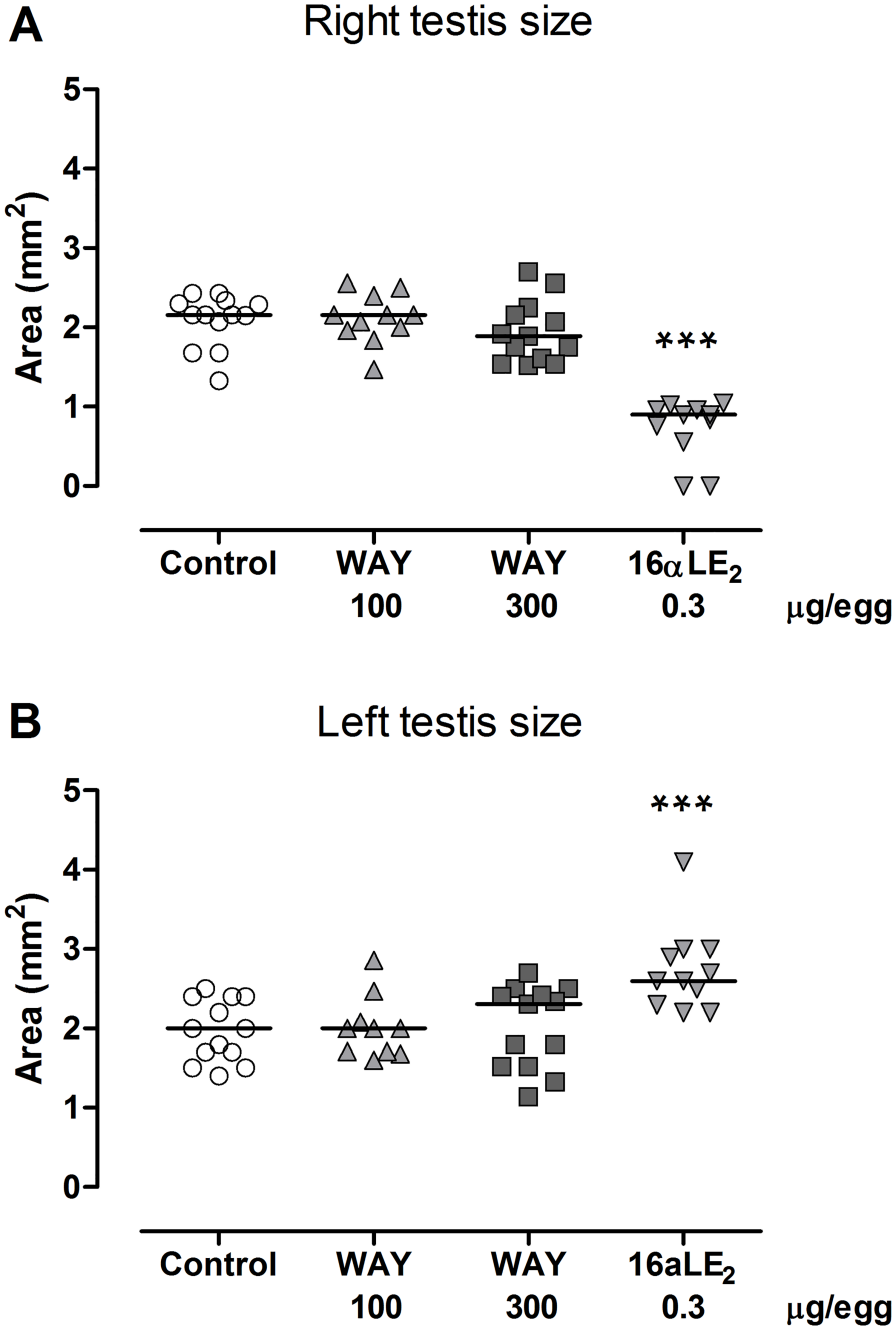
Size (length × width) of right (A) and left (B) testis in male quail embryos. Embryos were treated by *in ovo* injection on E3 with vehicle, the ERβ agonist WAY, or the ERα agonist 16αLE_2_. Effects were assessed on E16. 16αLE_2_ significantly reduced the size of the right testis and increased the size of the left testis. This indicates a feminizing effect since in control females only the left ovary develops whereas the right ovary regresses. The horizontal lines indicate the medians. The number of examined males was 13, 11, 13 and 11 in the groups from left to right in the figures. Differences were analyzed using ANOVA (p < 0.0001, F = 40.0, total df = 47 in A; p = 0.0006, F = 7.1, total df = 46 in B). Following the ANOVA, all treated groups were compared with the control using Dunnett’s multiple comparison test.(***p <0.001).

**Fig 4:**
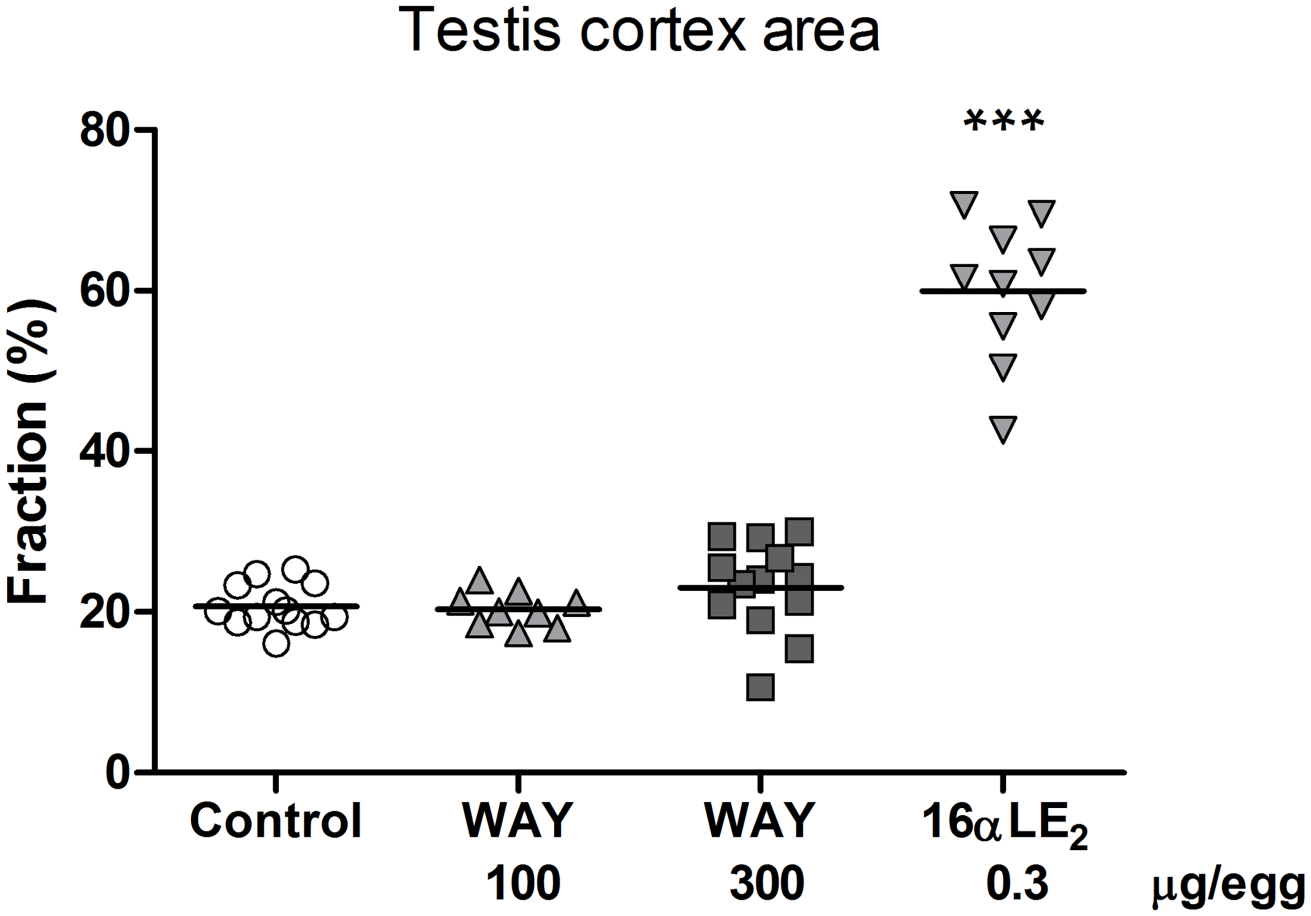
Fraction cortex in histological sections of the left testis in quail embryos. Embryos were treated by *in ovo* injection on E3 with vehicle, the ERβ agonist WAY, or the ERα agonist 16αLE_2_. Effects were assessed on E16. 16αLE_2_ caused growth of a thick ovary-like cortex containing oocyte-like germ cells. The horizontal line indicates the group mean. Differences in testis cortex area were analyzed using Kruskal-Wallis ANOVA (p < 0.0001 and H = 25.4), followed by Dunn's multiple comparison test. Asterisks indicate significant differences compared with the control (***p <0.001).

In all female embryos the left ovary was well developed, i.e. it is wider and more flat than a normal testis at the same developmental stage, and the right ovary was completely or almost completely regressed. In male embryos exposed to vehicle or WAY, the right and left testis appeared normal and were of equal size. By contrast, 16αLE_2_ induced feminization of the testes; the right testis was significantly reduced in size and the left testis was enlarged (Fig 3) and had an ovary-like shape. The formation of ovarian tissue in the left testis by treatment with 16αLE_2_ was confirmed by histology. In resemblance with an ovarian cortex, the cortex of the left testis was thick (Fig 4.) and contained oocyte-like germ cells.

### Hatching and growth

Birds exposed *in ovo* to vehicle, WAY, 16αLE_2_, or a mixture of WAY and 16αLE_2_ were raised and examined for persistent effects. Doses, hatching frequencies and number of examined birds are indicated in Table 2. The hatching frequency for embryos that hatched on E17 to E20 was significantly reduced by the combination of WAY and 16αLE_2_ but was not affected by each substance alone. Body weights of male and female juveniles at one, two and three weeks after hatching did not differ between groups and there were no weight differences between the adult males of the different groups (S1 Data).

**Table 2:** Dose, hatching frequency and number of examined juvenile and adult quails.

Treatment group	Dose (μg/egg)	Hatching frequency	Juveniles		Adult Males
			Females	Males	
Control	0	47/72	9	11	9
WAY	100	38/73	14	13	9
16αLE_2_	0.3	39/72	10	12	8
WAY+16αLE_2_	0.3+100	33/72*	14	7	9

*One-sided Fisher's exact test, p<0.05.

### Oviducts

#### Juvenile females

**Fig 5:**
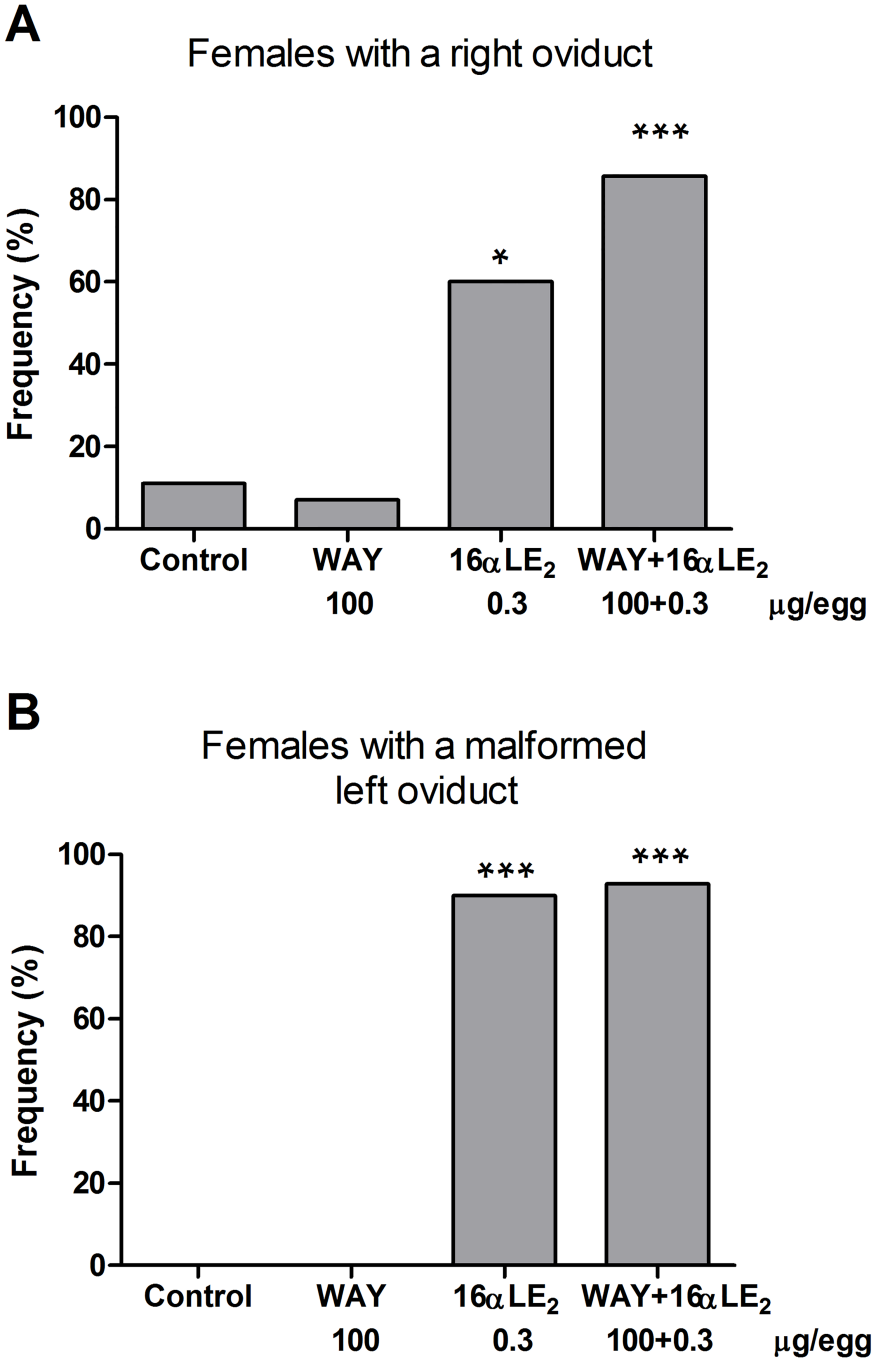
Frequencies of a partially developed right oviduct (A) and a malformed left oviduct (B) in juvenile female quails. A right oviduct longer than 10 mm was defined as partially developed. The birds were treated *in ovo* on E3 and effects were assessed three weeks after hatching. The number of examined females was 9, 14, 10 and 14 in the control, WAY, 16αLE_2_ and 16αLE_2_ + WAY combination groups, respectively. Frequencies in the treated groups were compared with the frequency in the control using one-sided Fisher's exact test (*p <0.05; ***p <0.001).

In all control and WAY-treated 3-week-old females, the left oviduct was well developed and only small remnants of the right oviduct were found. Only one control female and one female in the WAY group had a right oviduct that was longer than 10 mm; these were 12 mm and 13 mm, respectively. The frequency of juvenile females with a partially developed right oviduct (>10mm) was significantly increased in the groups treated with 16αLE_2_ or the mixture of WAY and 16αLE_2_ (Fig 5A). The vast majority of females in these two groups displayed gross malformations of the oviducts (Fig 5B). Typically, one or both oviducts were abnormally thick, contained a white cheese-like substance, and frequently had a liquid-filled cyst at the cranial end.

#### Juvenile and adult males

**Fig 6:**
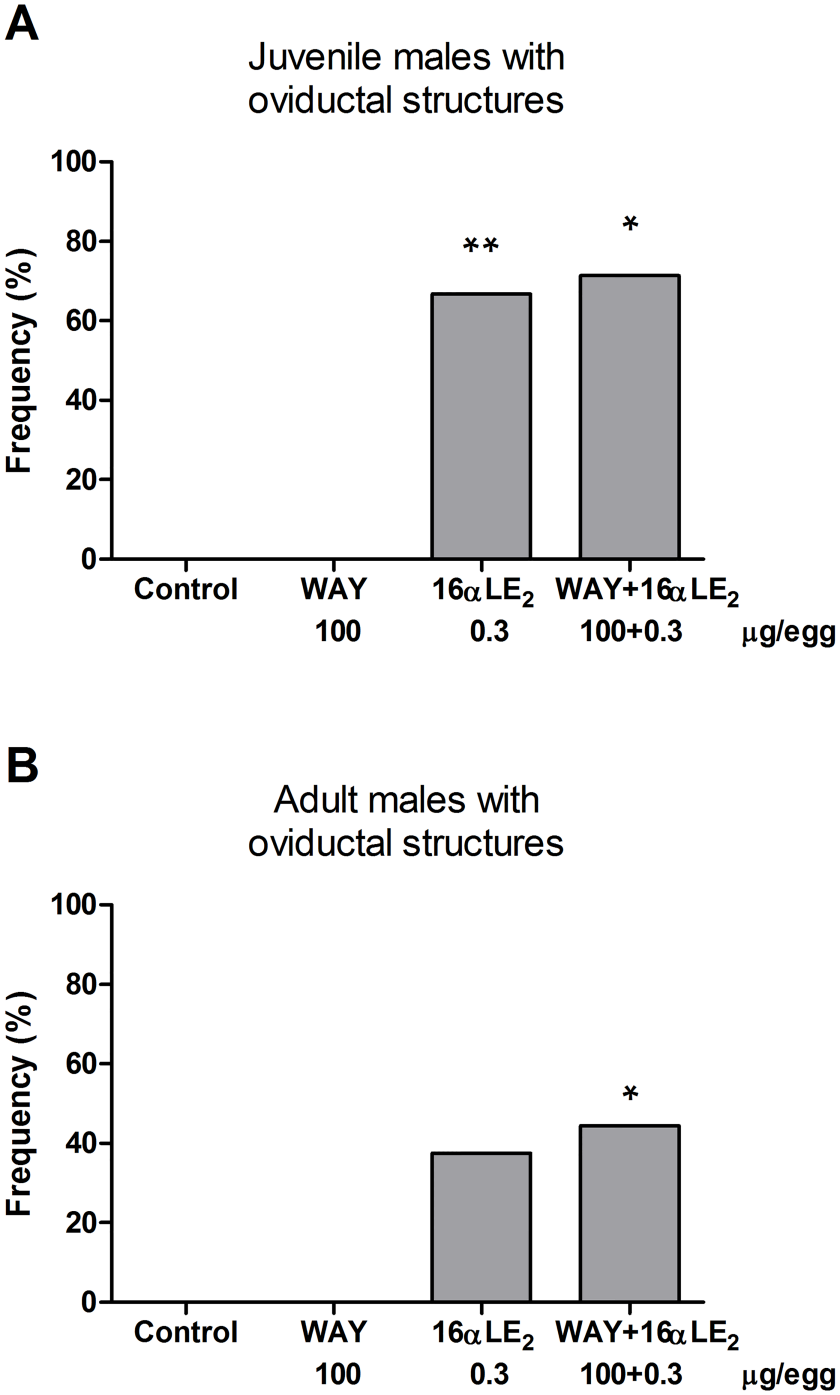
Frequencies of juvenile (A) and adult (B) male quails with oviductal structures. In the majority of the cases, the oviducts consisted of one or more large liquid-filled cysts. The birds were treated *in ovo* on E3 and effects were assessed in juveniles three weeks after hatching and in adult males nine weeks after hatching. The number of examined juvenile males was 11, 13, 12 and 7 in the control, WAY, 16αLE_2_ and 16αLE_2_ + WAY combination groups, respectively. Nine adult males were examined from each group except for the 16αLE_2_ group from which 8 males were examined. Frequencies in the treated groups were compared with the frequency in the control using one-sided Fisher's exact test (*p <0.05; **p<0.01).

Control and WAY-treated juvenile and adult males had no oviductal structures in the abdominal cavity. The majority of juvenile males exposed to 16αLE_2_ or to the combination of 16αLE_2_ and WAY displayed grossly malformed oviductal structures on one or both sides (Fig 6A). Such structures were seen also among the adult males in these groups although at lower frequencies (Fig 6B). The oviduct malformations were generally more severe in males than in females of the same group and often the whole oviduct consisted of one large or several smaller cysts. The white cheese-like substance was not as common in males as in females.

### Testes and cloacal gland

**Fig 7:**
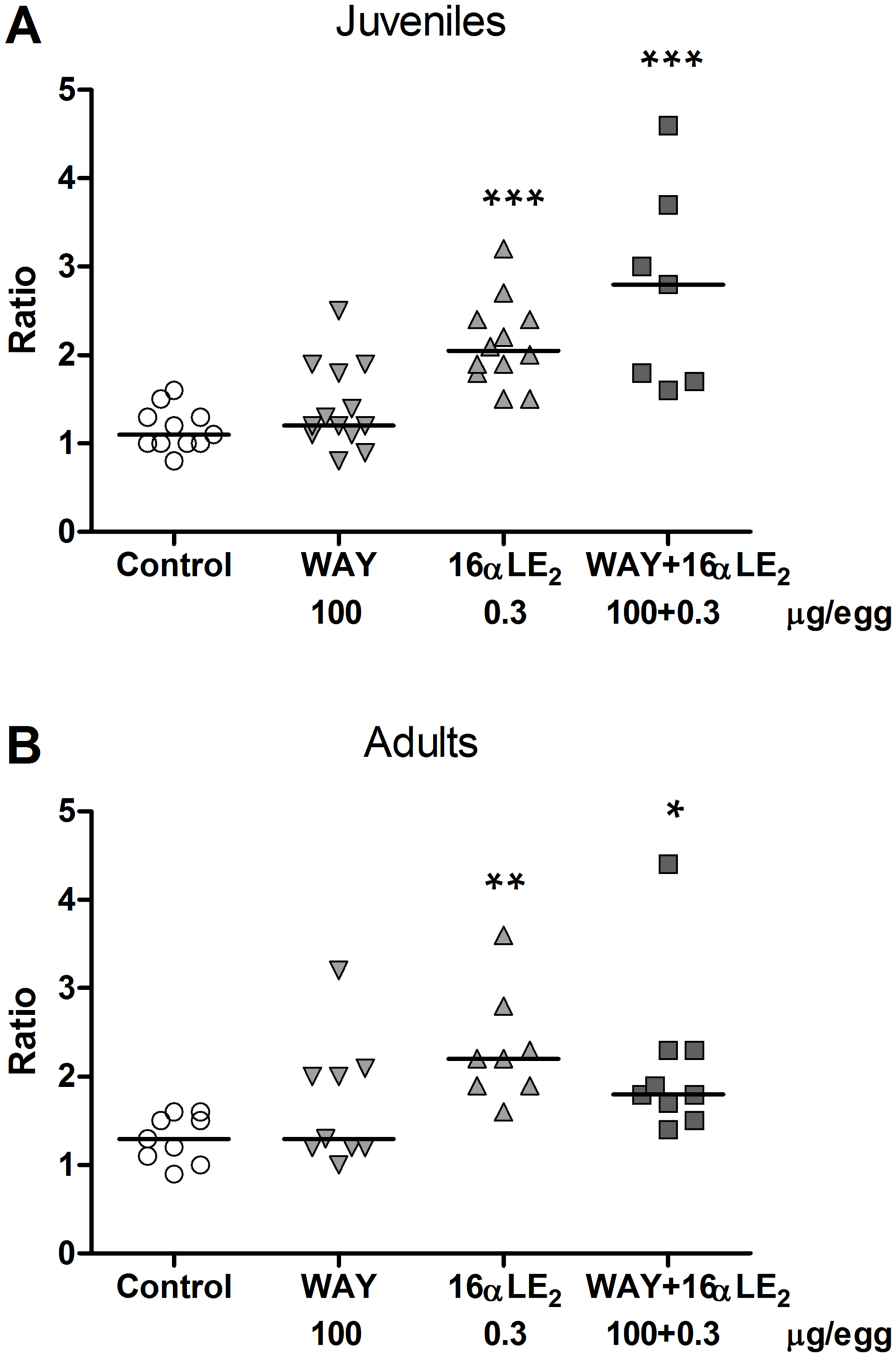
Testis weight ratio (left/right) in juvenile (A) and adult (B) male quails. The birds were treated *in ovo* on E3 with vehicle, the ERβ agonist WAY, the ERα agonist 16αLE_2_, or with both compounds in combination. Ratios were determined in juveniles three weeks after hatching and in adult males nine weeks after hatching. The horizontal lines indicate the medians. Differences in ratios were analyzed using ANOVA. (p < 0.0001, F = 16.0, total df = 42 in A; p = 0.0026, F = 5.9, total df = 34 in B). Following the ANOVA, all treated groups were compared with the control using Dunnett’s multiple comparison test (*p <0.05; **p<0.01; ***p <0.001).

**Fig 8:**
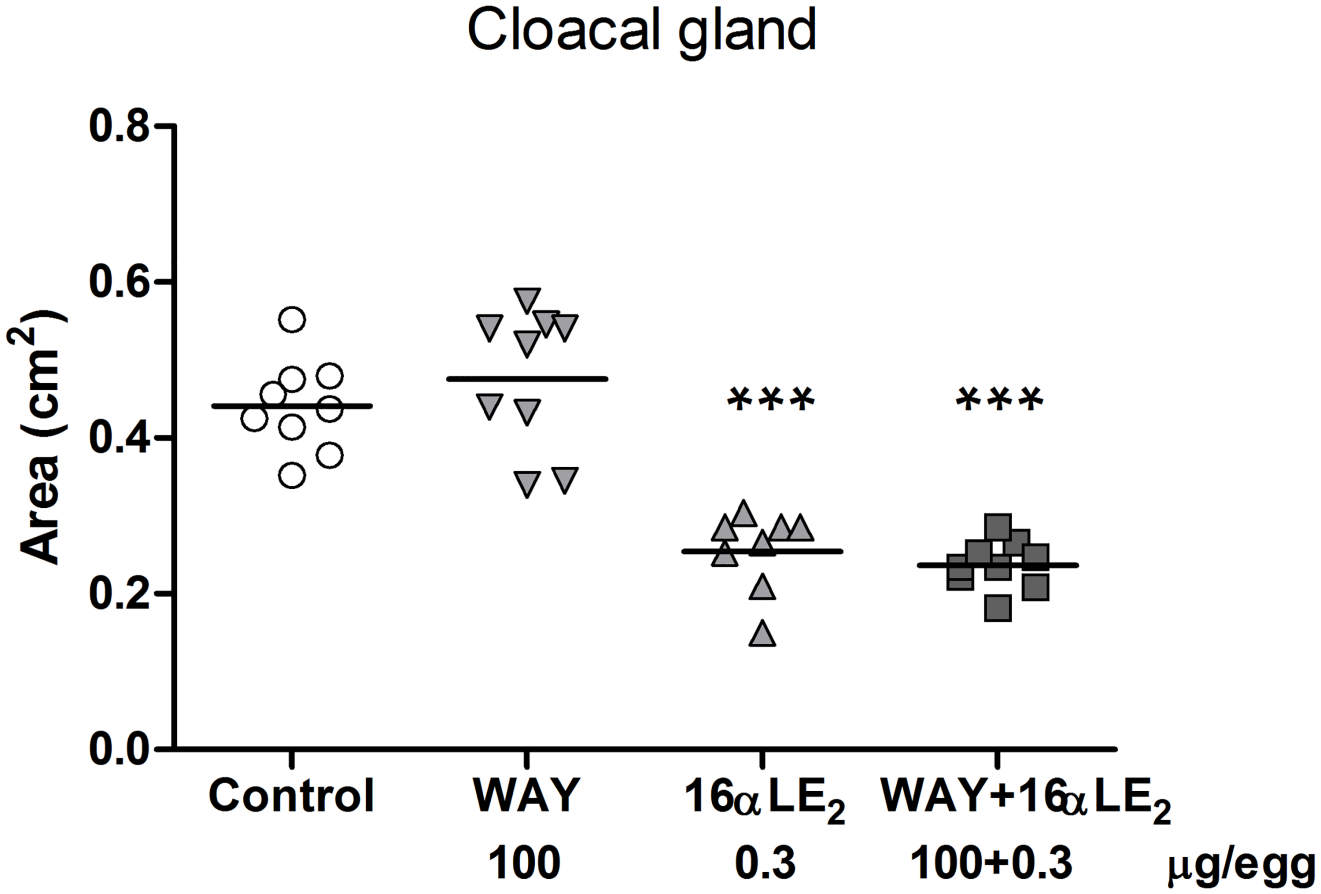
Cloacal gland area in adult male quails. The birds were treated *in ovo* on E3 with vehicle, the ERβ agonist WAY, the ERα agonist 16αLE_2_, or with both compounds in combination. Cloacal gland areas were measured nine weeks after hatching. The horizontal lines indicate the means. Differences were analyzed using ANOVA (p < 0.0001, F = 36.0 and df = 32). Following the ANOVA, all treated groups were compared with the control using Dunnett’s multiple comparison test (***p <0.001).

The testes of the juvenile males generally appeared normal in all groups. Exceptions were some dark spots on the testes which occurred in all groups (1–2 cases in each group) and one completely black testis found in a male treated with the combination of 16αLE_2_ and WAY. All control and WAY-treated adult males displayed normally appearing testes whereas deformed testes were found in four out of eight 16αLE_2_-treated males (p<0.05) and in three out of nine males in the combination group (not significant). The observed deformities included irregular shape, granulated surface and discoloration. In the 16αLE_2_ group, one adult had two small epididymides and another lacked epididymis on both sides. In the combination group one adult male displayed a misshapen left epididymis. The organs showing abnormal appearance were not further examined. The gonado-somatic index (testis weight / body weight) was not affected by treatment in neither juvenile nor adult males (S1 Data). The testis weight ratio (left testis weight / right testis weight) was significantly increased in both juvenile and adult males treated with 16αLE_2_ or 16αLE_2_ and WAY in combination, compared with the control (Fig 7A and 7B). The cloacal gland area was measured in adult males only and was found to be significantly decreased by 16αLE_2_ and by the combination of 16αLE_2_ and WAY (Fig 8).

### Sexual behavior and plasma testosterone

**Fig 9:**
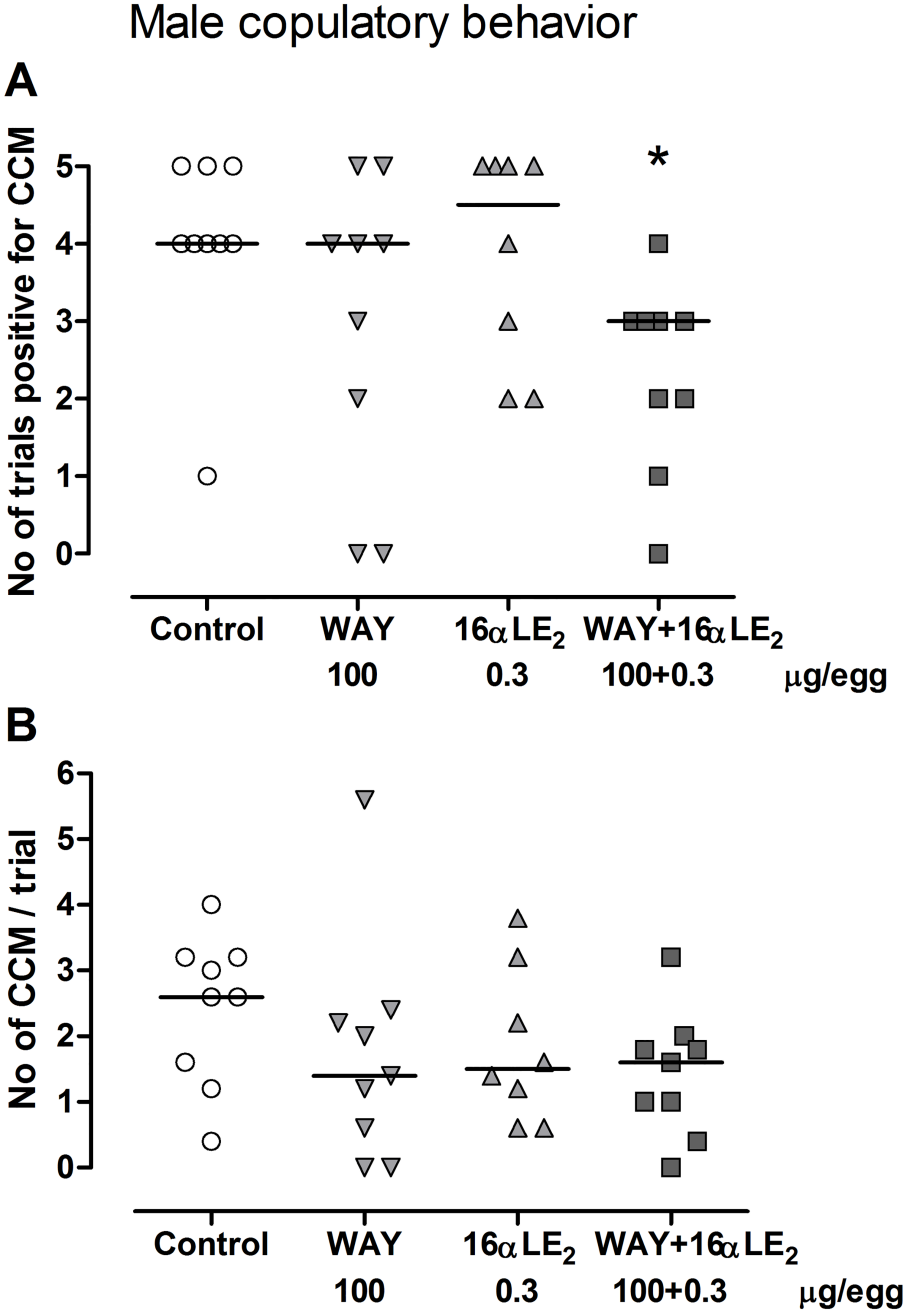
Copulatory behavior displayed by adult male quails. For each bird, the number of trials out of five in which cloacal contact movements (CCM) were displayed at least once is shown in A and the average number of times that each bird showed this behavior across the five trials is shown in B. The birds were treated *in ovo* on E3 with vehicle, the ERβ agonist WAY, the ERα agonist 16αLE_2_, or with both compounds in combination. The behavior was assessed once a day for five consecutive days during the ninth week after hatching. The horizontal lines indicate the group medians. Differences in behavior were analyzed using Kruskal-Wallis ANOVA (p = 0.0476, H = 7.9 in A; p = 0.30, H = 3.7 in B). Following the Kruskal-Wallis ANOVA, all treated groups were compared with the control using Dunn’s multiple comparison test (*p <0.05).

Each behavior in the male copulatory sequence was scored as the number of trials out of five possible where the behavior was displayed by the male at least once. The number of times that cloacal contact movements were displayed during a trial was also noted. Neck grab, mount attempt and mount were not significantly affected by treatment. All males performed neck grab and mount attempt at all five test occasions, except one WAY-treated male that did not show mount attempt at the first test occasion (S1 Data). Males in all groups performed rather well also in mounting the female and most showed a successful mount at least at four out of five test occasions. Exceptions were one WAY-treated male that never mounted and one male treated with the combination of WAY and 16αLE_2_ that only mounted at two of the five test occasions. The fraction of the five trials in which cloacal contact movements were displayed at least once was significantly reduced by the combination of WAY and 16αLE_2_ (Fig 9A). However, the average number of times that each bird showed this behavior across the five trials was not affected by treatment (Fig 9B).

**Fig 10:**
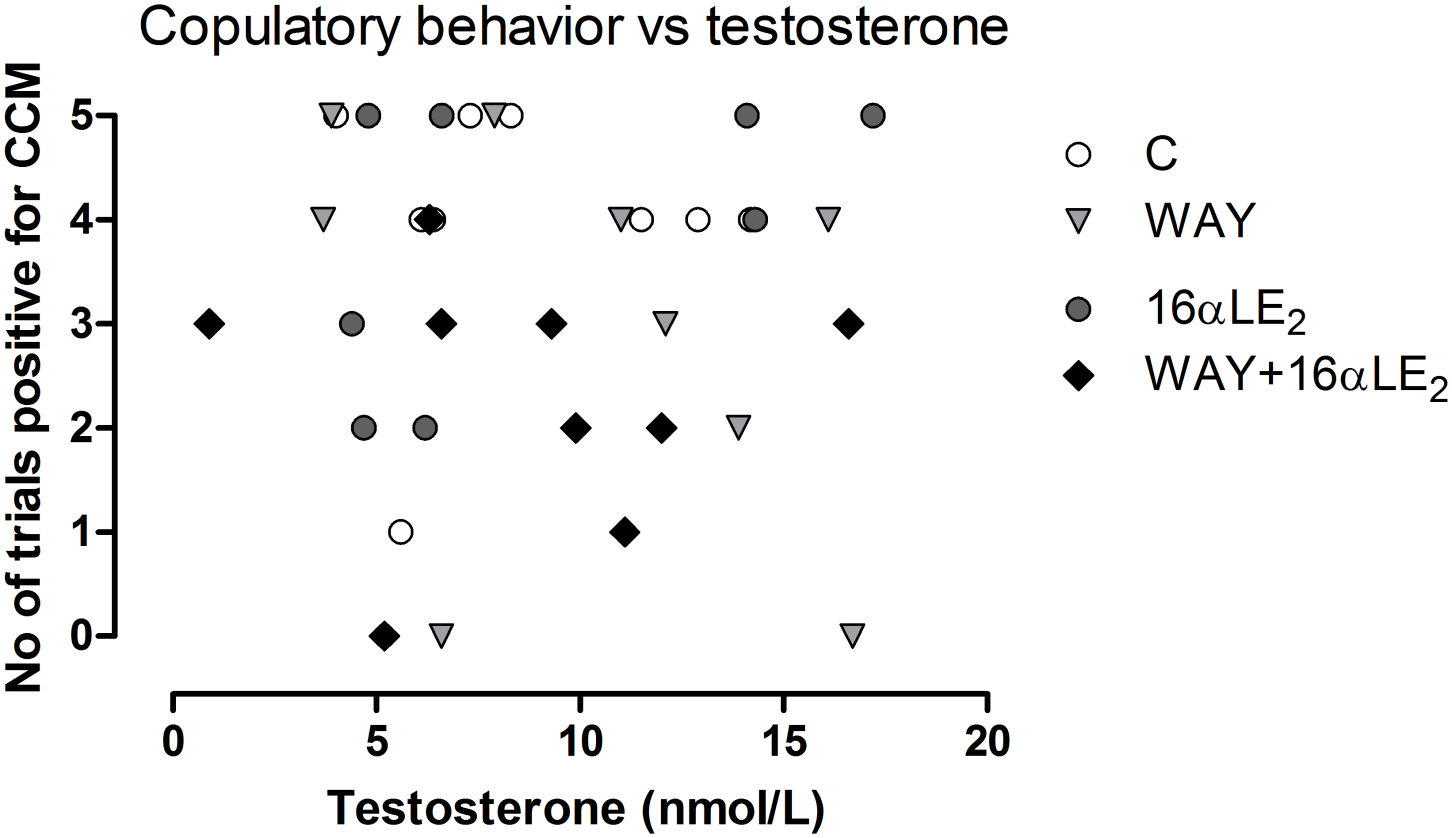
Copulatory behavior vs plasma testosterone concentration in adult males. The copulatory behavior was scored as the number of trials out of five in which cloacal contact movements (CCM) were displayed at least once. Linear regression analyses revealed no correlation between testosterone concentration and copulatory behavior, and ANOVA analysis showed no difference in testosterone concentration between the groups.

The plasma testosterone concentration was not affected by treatment in males but was significantly lower in females than in males (p<0.001). The concentration (mean ± SD; nmol/L) of the control females was 1.3 ± 0.6, of the control males 8 ± 4, of the WAY-treated males 10 ± 5, of the 16αLE_2_-treated males 9 ± 5, and of the males treated with WAY and 16αLE_2_ in combination 9 ± 5. Any correlation between plasma testosterone concentration and copulatory behavior was analyzed both within each group and with all groups combined. Correlation was analyzed both in terms of the number of tests where CCM was displayed at least once and the number of CCM displayed per trial. We found no significant correlations. A scatter plot of copulatory behavior against plasma testosterone concentration is shown in Fig 10. The individuals with the lowest testosterone concentrations performed well in the behavior test. The results suggest that the reduced behavior seen in the combination group was not due to insufficient levels of activating testosterone.

## Discussion

In the present study we explored the roles of ERα and ERβ in estrogen-induced disruption of avian sex differentiation by exposing Japanese quail embryos to the ERα-selective agonist 16αLE_2_ and the ERβ-selective agonist WAY. Reproductive organ development was affected by the ERα agonist, but not by the ERβ agonist. The male copulatory behavior was not affected by either of the ER agonists when administered alone, but was slightly suppressed by a combination of the two compounds.

In birds, gonadal estrogens act during critical periods of development to promote female sexual differentiation of reproductive organs and behavior and therefore embryonic exposure to estrogenic compounds may disrupt sex differentiation of the reproductive organs and demasculinize reproductive behaviors as manifested later in life [41]. The quail eggs in the present study were injected on E3 and the embryos were consequently exposed via uptake from the yolk during the critical periods of differentiation of gonads [1] and copulatory behavior [5, 11].

In females, the ERα agonist 16αLE_2_ caused abnormal development of the embryonic Müllerian ducts and in juveniles malformations of the oviducts were found on both right and left side (the right oviduct is normally not developed). In males, the ERα agonist caused anomalous development of oviductal structures, decreased size of the cloacal gland and feminized testes. In male embryos, the left testis was ovary-like in shape (flattened), size (large) and histological appearance (thick cortex with oocyte-like germ cells). The right testis was reduced in size which partly resembles the strong regression of the right ovary seen in control females. Testicular deformities were found in adult males exposed to the ERα agonist and the left / right testis weight ratio was high compared with the control. Such effects on the reproductive organs have previously been described in for instance chicken, quail and gulls following embryonic exposure to excess estrogen or estrogenic compounds [2, 14–18, 42]. The results of excessive ERα stimulation on reproductive organ development in the present study confirm our previous findings from embryonic treatment of quail and chicken with 16αLE_2_ and another selective ERα-agonist, PPT [28, 33–35]. We found no effects on the studied reproductive organs following treatment with the ERβ agonist WAY, and the combination of 16αLE_2_ and WAY caused a response comparable to that of 16αLE_2_ alone. In accordance, the ERβ agonist DPN did not affect reproductive organ development in chicken embryos [35]. Together our results suggest that excessive activation of ERα but not of ERβ can result in similar anomalous development of reproductive organs as shown following exposure to various xenoestrogens. Further, the feminizing / demasculinizing effects in males suggest that ERα, and not ERβ, is involved in normal sex differentiation of ovary, Müllerian ducts / oviducts and cloacal gland in female quail. In accordance with our results, American alligators showed male-to-female sex reversal and Müllerian duct hyperplasia at a male-producing temperature when exposed to estradiol or the ERα-agonist PPT during sex determination, but not when exposed to WAY [43].

Our previous studies have shown that male copulatory behavior in quail is efficiently suppressed by exposure to non-selective ER agonists when administered from E3, whereas ERα-selective agonists do not affect this behavior at doses that markedly affect the reproductive organ differentiation [28, 33, 34]. Due to the lack of effect by ERα-activation we speculated that demasculinization of copulatory behavior requires activation of ERβ.

A putative role of ERβ in this process is further supported by that ERβ transcripts, but not ERα transcripts, were found in the embryonic quail brain on E9 (i.e. within the sensitive period) in nuclei that are implicated in male reproductive behaviors in adulthood [32]. In the present study we therefore explored the hypothesis that male copulatory behavior is demasculinized by estrogens via ERβ or via combined activation of ERα and ERβ. Our results showed that neither the ERα agonist 16αLE_2_ (0.3 μg/egg) nor the ERβ agonist WAY (100 μg/egg) significantly suppressed the behavior when tested alone. When administered together these substances significantly reduced the number of tests where cloacal contact movements were shown (Fig 9A). It has to be emphasized though that this effect by the combined exposure to the two compounds was fairly weak compared with their effects on the reproductive organs. Moreover, other behaviors in the copulatory sequence and the mean number of times that CCM was shown during the five trials were not significantly affected.

The modest effect on the copulatory behavior is likely not a consequence of limited uptake of the substances to the brain as both16αLE_2_ and WAY are known to cross the blood-brain barrier in rodents and exert effects in the brain [44–47]. Possibly WAY did not sufficiently activate ERβ in the quail; there is no experimental evidence of transactivation of quail ERβ by WAY and there is no known effect by embryonic activation of ERβ in quail that can be used as a positive control. However, the conserved amino acid residues in the ligand binding pocket of human and quail ERs suggest that WAY is ERβ-specific also in quail (S1 Fig).

## Conclusions

The results from our present and previous studies suggest that ERα, but not ERβ, is involved in female differentiation of the ovary and Müllerian duct/oviduct in birds and that exogenous compounds that activate ERα may disturb this process and cause abnormal development of the reproductive organs in both sexes. The modest effect on male copulatory behavior by the ERα and ERβ agonists, separately and in combination, suggests that the organizational effects of estrogens on this behavior during development is mainly mediated by other estrogen-responsive pathways than those activated by ERα and ERβ.

## Supporting information

S1 Fig Alignment of the human and Japanese quail ERα and ERβ protein sequences. The ruler shows the amino acid residue positions in the human ERα (hERα). The highlights indicate the position of the two amino acids residues that differ between hERα and hERα in the ligand binding pocket; these are hERα Leu_384_ (*) and Met_421_ (#) which correspond to hERβ Met_336_ and Ile_373_, respectively. The sequence alignment shows that the amino acid residues in both these positions are conserved between hERα and quail ERα (qERα) whereas hERβ Met_336_ (*) is replaced by a leucine residue in qERβ. The selectivity of 16αLE2 for ERα and of WAY for ERβ is however conferred by the hERα Met_421_ /hERβ Ile_373_ (#) residue substitution which is conserved between the two species. The protein sequences were retrieved from NCBI and aligned using BioEdit Sequence Alignment Editor. The accession numbers are: P03372.2 for hERα, AAN63674.1 for qERα, NP_001428.1 for hERβ and O93511.2 for qERβ.
(DOCX)

S1 Data Body weights, morphology, behavior and plasma testosterone concentrations in embryonic, juvenile and adult Japanese quail following embryonic treatment with selective estrogen receptor agonists. (XLSX)

S1 File ARRIVE Guidelines checklist. (DOCX)
