# Supplementary material for: Effects of selective and combined activation of estrogen receptor α and β on reproductive organ development and sexual behaviour in Japanese quail (Coturnix japonica)
Source: PLoS One. 2017 Jul 3;12(7):e0180548. doi: 10.1371/journal.pone.0180548 (PMC5495399; doi:10.1371/journal.pone.0180548)
Supplement: S1 Fig — The ruler shows the amino acid residue positions in the human ERα (hERα). The highlights indicate the position of the two amino acids residues that differ between hERα and hERα in the ligand binding pocket; these are hERα Leu384 (*) and Met421 (#) which correspond to hERβ Met336 and Ile373, respectively. The sequence alignment shows that the amino acid residues in both these positions are conserved between hERα and quail ERα (qERα) whereas hERβ Met336 (*) is replaced by a leucine residue in qERβ. The selectivity of 16αLE2 for ERα and of WAY for ERβ is however conferred by the hERα Met421 /hERβ Ile373 (#) residue substitution which is conserved between the two species. The protein sequences were retrieved from NCBI and aligned using BioEdit Sequence Alignment Editor. The accession numbers are: P03372.2 for hERα, AAN63674.1 for qERα, NP_001428.1 for hERβ and O93511.2 for qERβ. (DOCX) [file pone.0180548.s001.docx]

**S1 Figure**

10 20 30 40 50 60 70

....|....| ....|....| ....|....| ....|....| ....|....| ....|....| ....|....|

hERα MTMTLHTKAS GMALLHQIQG NELEPLNRPQ LKIPLERPLG EVYLDSSKPA VYNYPEGAAY EFNAAAAANA

qERα MTMTLHTKAS GVTLLHQIQG TELETLSRPQ LKIPLERSLS DMYVESNKTG VFNYPEGATY DFG----TTA

hERβ --MDIKNSPS --------SL NSPSSYNCSQ SILPLEH--G SIYIPSSYVD SHHEYP---- --------AM

qERβ ---------- ---------- ---------- ---------- ---------- ---------- ---------M

80 90 100 110 120 130 140

....|....| ....|....| ....|....| ....|....| ....|....| ....|....| ....|....|

hERα QVYGQTGLPY GPGSEAAAFG SNGLGGFPPL NSVSPSPLML LHPPPQLSPF LQPHGQQVPY YLENEPSGYT

qERα PVYGSTTLSY APTSES--FG SSSLAGFHSL NNVPPSPVVF LQTAPQLSPF IHHHSQQVPY YLENEQGSFG

hERβ TFYSPAVMNY SIPSNVTNLE GGPGR----- --QTTSPNVL WPTPGHLSP- LVVHRQLSHL YAEPQKSPWC

qERβ AFCSPAMMNY NIASNFGDSE SASVR----- --QTSSPSLL WSAPGHLSP- LTLHCQLSLL YAEQPKSPWC

150 160 170 180 190 200 210

....|....| ....|....| ....|....| ....|....| ....|....| ....|....| ....|....|

hERα VREAGPPAFY RPNSDNRRQG GRERLASTND KGSMAMESAK ETRYCAVCND YASGYHYGVW SCEGCKAFFK

qERα MRETAPPAFY RPSSDNRRHS IRERMSSASE KGSLSMESTK ETRYCAVCND YASGYHYGVW SCEGCKAFFK

hERβ EARSLEHTLP VNRETLKRKV SGNRCASPVT G----PGSKR DAHFCAVCSD YASGYHYGVW SCEGCKAFFK

qERβ EARPLEPVLP VSRETLKRKT NGSDCTSPIA SN---PGSKR DAHFCAVCSD YASGYHYGVW SCEGCKAFFK

220 230 240 250 260 270 280

....|....| ....|....| ....|....| ....|....| ....|....| ....|....| ....|....|

hERα RSIQGHNDYM CPATNQCTID KNRRKSCQAC RLRKCYEVGM MKGGIRKDRR GGRMLKHKRQ RDDGEGRGEV

qERα RSIQGHNDYM CPATNQCTID KNRRKSCQAC RLRKCYEVGM MKGGIRKDRR GGRMMKQKRQ REEQESRNGE

hERβ RSIQGHNDYI CPATNQCTID KNRRKSCQAC RLRKCYEVGM VKCGSRRERC GYRLVRRQRS ADEQLHCAGK

qERβ RSIQGHNDYI CPATNQCTID KNRRKSCQAC RLRKCYEVGM MKCGSRRERC GYRILRRHRN SED---CMGK

290 300 310 320 330 340 350

....|....| ....|....| ....|....| ....|....| ....|....| ....|....| ....|....|

hERα GSAGDMRAAN LWPSPLMIKR SKKNSLALSL TADQMVSALL DAEPPILYSE YDPTRPFSEA SMMGLLTNLA

qERα ASSTELRAPT LWTSPLVVKH NKKNSPALSL TAEQMVSALL EAEPPIVYSE YDPNRPFNEA SMMTLLTNLA

hERβ AKRSGGHAP- ---------- RVRELLLDAL SPEQLVLTLL EAEPPHVLIS R-PSAPFTEA SMMMSLTKLA

qERβ TKKYNEAAT- ---------- RVKEILLSTV SPEQFVLTLL EAEPPNVLVS R-PSKPFTEA SMMMSLTKLA

360 370 380 390 400 410 420

....|....| ....|....| ....|....| ...*****|....| ....|....| ....|....| ....|....|

hERα DRELVHMINW AKRVPGFVDL TLHDQVHLLE CAWLEILMIG LVWRSMEHPG KLLFAPNLLL DRNQGKCVEG

qERα DRELVHMINW AKRVPGFVDL TLHDQVHLLE CAWLEILMIG LVWRSMEHPG KLLFAPNLLL DRNQGKCVEG

hERb DKELVHMISW AKKIPGFVEL SLFDQVRLLE SCWMEVLMMG LMWRSIDHPG KLIFAPDLVL DRDEGKCVEG

qERb DKELVHMIGW AKKIPGFIDL SLYDQVRLLE SCWLEVLMIG LMWRSIDHPG KLIFAPDLVL DRDEGKCVEG

430 440 450 460 470 480 490

**#**...|....| ....|....| ....|....| ....|....| ....|....| ....|....| ....|....|

hERα MVEIFDMLLA TSSRFRMMNL QGEEFVCLKS IILLNSGVYT FLSSTLKSLE EKDHIHRVLD KITDTLIHLM

qERα MVEIFDMLLA TAARFRMMNL QGEEFVCLKS IILLNSGVYT FLSSTLKSLE ERDYIHRVLD KITDTLIHFM

hERβ ILEIFDMLLA TTSRFRELKL QHKEYLCVKA MILLNSSMYP LVTATQDADS SR-KLAHLLN AVTDALVWVI

qERβ ILEIFDMLLA MTSRFRELKL QHKEYLCVKA MILLNSSMFP LS--AEEPES NR-KLHHLLN VVTDALVWVI

500 510 520 530 540 550 560

....|....| ....|....| ....|....| ....|....| ....|....| ....|....| ....|....|

hERα AKAGLTLQQQ HQRLAQLLLI LSHIRHMSNK GMEHLYSMKC KNVVPLYDLL LEMLDAHRLH APTSRGGASV

qERα AKSGLSLQQQ HRRLAQLLLI LSHIRHMSNK GMEHLYNMKC KNVVPLYDLL LEMLDAHRLH APAARSAAPM

hERβ AKSGISSQQQ SMRLANLLML LSHVRHASNK GMEHLLNMKC KNVVPVYDLL LEMLNAHVLR G-CKSSITGS

qERβ AKSGIPSQQQ TTRLANLLML LSHVRHASNK GMEHLLSMKC KNVVPVYDLL LEMLNAHTLR GQRKSPVTHP

570 580 590

....|....| ....|....| ....|....| ....|.

hERα EETDQSHLAT AGSTSSHSLQ KYYITG-EAE GFPATV

qERα EEENRSQLTT A-PASSHSLQ SFYINSKEEE SMQNTI

hERβ ECSPAEDSKS KEGSQNPQSQ ---------- ------

qERβ DFEQVSHFQV ---------- ---------- ------

**S1 Figure. Alignment of the human and Japanese quail ERα and ERβ** **protein sequences.** The ruler shows the amino acid residue positions in the human ERα (hERα). The highlights indicate the position of the two amino acids residues that differ between hERα and hERβ in the ligand binding pocket; these are hERα Leu_384_ (*) and Met_421_ (#) which correspond to hERβ Met_336_ and Ile_373_, respectively. The sequence alignment shows that the amino acid residues in both these positions are conserved between hERα and quail ERα (qERα) whereas hERβ Met_336_ (*) is replaced by a leucine residue in qERβ. The selectivity of 16αLE2 for ERα and of WAY for ERβ is however conferred by the hERα Met_421_ /hERβ Ile_373_ (#) residue substitution which is conserved between the two species. The protein sequences were retrieved from NCBI and aligned using BioEdit Sequence Alignment Editor. The accession numbers are: P03372.2 for hERα, AAN63674.1 for qERα, NP_001428.1 for hERβ and O93511.2 for qERβ.
